# Role of Non-Coding RNA in Neurological Complications Associated With Enterovirus 71

**DOI:** 10.3389/fcimb.2022.873304

**Published:** 2022-04-25

**Authors:** Feixiang Yang, Ning Zhang, Yuxin Chen, Jiancai Yin, Muchen Xu, Xiang Cheng, Ruyi Ma, Jialin Meng, Yinan Du

**Affiliations:** ^1^ School of Basic Medical Sciences, Anhui Medical University, Hefei, China; ^2^ Department of Urology, The First Affiliated Hospital of Anhui Medical University, Hefei, China; ^3^ Institute of Urology, Anhui Medical University, Hefei, China; ^4^ Anhui Province Key Laboratory of Genitourinary Diseases, Anhui Medical University, Hefei, China; ^5^ First School of Clinical Medicine, Anhui Medical University, Hefei, China; ^6^ School of Public Health, Anhui Medical University, Hefei, China

**Keywords:** virus-host interaction, enterovirus 71, hand, foot, and mouse disease, microRNA, long non-coding RNA, non-coding RNA

## Abstract

Enterovirus 71 (EV71) is the main pathogenic virus that causes hand, foot, and mouth disease (HFMD). Studies have reported that EV71-induced infections including aseptic meningitis, acute flaccid paralysis, and even neurogenic pulmonary edema, can progress to severe neurological complications in infants, young children, and the immunosuppressed population. However, the mechanisms through which EV71 causes neurological diseases have not been fully explored. Non-coding RNAs (ncRNAs), are RNAs that do not code for proteins, play a key role in biological processes and disease development associated with EV71. In this review, we summarized recent advances concerning the impacts of ncRNAs on neurological diseases caused by interaction between EV71 and host, revealing the potential role of ncRNAs in pathogenesis, diagnosis and treatment of EV71-induced neurological complications.

## 1 Introduction

Enteroviruses (EVs) are a genus of the Picornaviridae family characterized by small, single-stranded, positive-sense RNA (Solomon et al., 2010; Baggen et al., 2018). There are 13 species in this family, of which 7 species, including four species of enteroviruses (enteroviruses A, B, C, and D) and three species of rhinoviruses (rhinoviruses A, B, and C) are pathogenic to humans (Nikonov et al., 2017). Enterovirus 71 (EV71) is a member of species group A and has an icosahedral structure that is characteristic of all EVs. The viral capsid comprises 60 repeating units referred to as protomers. Each protomer consists of four structural viral proteins (surface proteins (VP1-VP3) and the internal protein (VP4)) (Solomon et al., 2010; Plevka et al., 2012; Baggen et al., 2018). The P1 coding region of the virus genome codes for structural proteins, whereas other seven non-structural proteins (proteins 2A-2C and 3A-3D) are encoded by the P2 and P3 regions (Solomon et al., 2010). Structure proteins of EV71 play important roles in viral pathogenicity, virulence and host resistance, as well as serve as regulatory targets for biological factors (Zheng et al., 2011; Wang B. et al., 2013).

EV71 is the main etiological agent that causes brief, generally mild, self-limiting HFMD, which is characterized by red spots or herpes on the hands, feet, and mouth and which resolves in 3-7 days without treatment (Solomon et al., 2010; Cox and Levent, 2018). Since EV71 was first isolated from the human central nervous system in 1974 (Schmidt et al., 1974). The EV71-associated neurological diseases, such as aseptic meningitis, acute flaccid paralysis, neurogenic cardiopulmonary failure and fatal encephalitis, have been widely reported in China, America, Brazil, Vietnam and other countries (Xing et al., 2014; Huang et al., 2015; Liu et al., 2015; Hasbun et al., 2017; B'Krong et al., 2018; Ramalho et al., 2019). A large epidemiological study conducted from 2008 to 2012 in China reported 7,200,092 probable cases among which 80% laboratory-confirmed severe cases (patients with neurological or cardiopulmonary complications) and 93% fatal cases were attributed to EV71 infection (Xing et al., 2014). Currently, there are no specific therapeutic options for EV71-induced neurological diseases, and the mechanisms of severe nervous system diseases have not been fully elucidated (Ooi et al., 2010; Solomon et al., 2010; Chen et al., 2020). Expanding evidence reveals that ncRNAs play essential roles in normal physiological and pathological processes (Beermann et al., 2016). Researchers found that ncRNAs were closely related to development of HFMD and pathogenicity of EV71, which may provide basis for pathogenesis, diagnosis and treatment of EV71-associated diseases.

## 2 Overview of Non-Coding RNAs

ncRNAs are RNAs without the potential for encoding biological proteins. Based on the number of nucleotides (nt), they are divided into two subclasses, small or short non-coding RNAs (less than 200 nt) and long non-coding RNAs (lncRNAs) (more than 200 nt) (Kapranov et al., 2007; Esteller, 2011; Beermann et al., 2016; Engreitz et al., 2016). Small non-coding RNAs are further classified into three main categories: microRNAs (miRNAs), short interfering RNAs (siRNAs), and piwi-interacting RNAs (piRNAs). Small non-coding RNAs act as disincentives to gene expression and regulation by combining with members of the Argonaute protein (Ago protein) superfamily (Carthew and Sontheimer, 2009). miRNAs, which mediate post-transcriptional gene suppression by binding mRNAs or viral genomes, are one of the most important and widely studied classes of ncRNAs (He and Hannon, 2004). In the nucleus, lncRNAs modulate expressions of neighboring genes through chromatin remodeling, and transcriptional and post-transcriptional regulation, thereby regulating biological processes (Mercer et al., 2009; Engreitz et al., 2016).

The synthesis of miRNAs is dependent on two pivotal enzymes, Drosha and Dicer, which belong to the ribonuclease-III (RNase III) family (Hutvágner et al., 2001; Lee et al., 2003). Primary miRNA (pri-miRNA) is transcribed from endogenous miRNA genes by RNA polymerase II (Pol II) to generate pre-miRNA after processing by Drosha inside the nucleus (Lee et al., 2003). Exportin-5 is involved in extranuclear transportation of pre-miRNA, which is subsequently cleaved by Dicer into an imperfect dsRNA duplex (miRNA: miRNA duplex) (Hutvágner et al., 2001; Carthew and Sontheimer, 2009). One miRNA strand: miRNA duplex is assembled into an RNA-induced silencing complex (RISC), namely miRISC. miRISC mediates post-transcriptional gene inhibition by translational repression or mRNA cleavage (He and Hannon, 2004; Rana, 2007; Carthew and Sontheimer, 2009). In various aspects, such as the same type of transcriptase, Pol II, lncRNAs are similar to mRNAs. However, compared to mRNAs, lncRNAs exhibit a lower transcription number and are evolutionarily conserved (Quinn and Chang, 2016). lncRNAs modulate genes expressions by interacting with chromatin and proteins through secondary structures such as hairpin and stem ring structures (Quinn and Chang, 2016; Statello et al., 2021), and this function plays an important role in the body against external infection.

ncRNAs have ability to cope with environmental changes and defend against external threats through the corresponding machinery. Dysregulated ncRNAs may damage various physiological processes and promote pathological conditions. For instance, dysregulated miR-143/145 cluster, which is extensively recognized as a tumor suppressor, promotes tumor growth by inducing angiogenesis in the tumor microenvironment (Dimitrova et al., 2016). The focus of this review is ncRNAs, particularly miRNAs and lncRNAs, with an emphasis on the effect of miRNAs on development of EV71-induced CNS complications and the potential of lncRNAs and miRNAs as biomarkers for clinical diagnosis and therapeutic targets.

## 3 Role of miRNAs in EV71-Induced CNS Infection

### 3.1 miRNAs and Neurotropism of EV71

Several studies on polioviruses (PVs), the one important species of enterovirus, have widely explored enterovirus tropism. Although viral tropism is determined by cellular receptors (Holland, 1961), internal ribosomal entry sites (IRESs) (Gromeier et al., 1996), and interference responses (especially α/β IFN) (Ida-Hosonuma et al., 2005), the cellular receptors play the most important role in cell and tissue tropism of PV. Previous studies have shown that non-susceptible mouse cells became susceptible after introducing human PVR gene into the mouse genome, and ultimately presented with CNS diseases similar to those in infected humans (Ren et al., 1990; Koike et al., 1991).

Relative to PVR, EV71 receptors are more complicated. Scavenger receptor class B, member 2 (SCARB2, also known as LGP85), which belongs to the CD36 family, is a type III transmembrane protein involved in membrane transport. SCARB2 is a major receptor for EV71 and plays a crucial role in attachment, internalization, and viral conformational changes for uncoating, which determines the cell and tissue tropism of EV71 (Yamayoshi et al., 2009; Dang et al., 2014). SCARB2 is highly expressed in several cells and tissues, including CNS neurons, pneumocytes, hepatocytes, splenocytes, renal tubular epithelia, and intestinal epithelia (Fujii et al., 2013). Fujii et al. (2013) and Yang et al. (2019) demonstrated that expression of only SCARB2was sufficient to allow transgenic mice to develop EV71-associated CNS diseases that resemble those in infected humans. Moreover, SCARB2 expression profiles in mice were comparable to those in humans, which may explain neurotropism and cell tropism of EV71. Although SCARB2 is of great significance in EV71 infection and tropism, it is not the only receptor that is implicated in EV71 infection. During EV71 infection process, other molecules can support attachment but not uncoating. These molecules are known as “attachment receptors” and they include P-selectin glycoprotein ligand-1 (PSGL-1) (Nishimura et al., 2009), annexin A2 (Anx2) (Yang et al., 2011), vimentin (Du et al., 2014), sialylated glycan (Yang et al., 2009), heparan sulfate glycosaminoglycan (Tan et al., 2013), nucleolin (Su et al., 2015), fibronectin (He et al., 2018) and prohibitin (Too et al., 2018). Attachment receptors lack the uncoating function, thus SCARB2 has a stronger correlation with EV71 infection processes compared to attachment receptors and is the decisive receptor that mediates EV71 cell and tissue tropism (He et al., 2014; Kobayashi and Koike, 2020).

miRNAs regulate EV71 tropism mainly by modulating the expression of SCARB2. Directly, miR-127-5p targets the SCARB2 mRNA 3’ untranslated region (UTR) and suppresses expression of SCARB2 in Gaucher fibroblasts (Siebert et al., 2014). Through further experiments, Feng et al. confirmed that miR-127-5p expression was upregulated after EV71 infection and that upregulation of miR-127-5p downregulated SCARB2 levels on cell surfaces through specific target binding, which principally affected the susceptibility of uninfected cells to EV71 infection and cell tropism of EV71 (Feng C. et al., 2017). Jin et al. found that downregulation of hsa-miR-3605-5p might advance tSCARB2 expression in human embryonic kidney 293T cells infected with coxsackievirus A16 (CVA16), thereby increasing susceptibility to EV71/CVA16 (Jin et al., 2017). Moreover, miR-202-3p (Li et al., 2020), miR-19a-5p (Siebert et al., 2014), and miR-1262 (Siebert et al., 2014) attenuated expression of SCARB2 mRNAs and proteins in non-EV71-indected cells, providing a basis for further research on EV71 infection and proliferation.

### 3.2 Effects of miRNAs on CNS Invasion of EV71

As a classic species of neurotropic enteroviruses transmitted by the fecal-oral route, EV71 proliferates in the digestive tract and invades the brain and other tissues and organs, resulting in encephalitis and other diseases (Ooi et al., 2010). After initial infection in the gastrointestinal tract neurotropic enteroviruses cross the blood-brain barrier (BBB) into the CNS through multiple routes. Intensive studies on CNS invasion routes of PVs have reported presence of three fundamental pathways through which enteroviruses gain access to the CNS (Huang and Shih, 2015; Chen et al., 2020). First, upon reaching the BBB by hematogenous transport, enteroviruses directly infect brain microvascular endothelial cells (BMECs) that constitute and maintain the integrity as well as permeability of the BBB. For instance, activation of the protein tyrosine phosphatase SHP-2 by PVR (Coyne et al., 2007) and attachment of PVs by mouse transferrin receptor 1 (Mizutani et al., 2016) facilitate PV CNS invasion by damaging BMECs. Second, enteroviruses hijack retrograde axonal transport [transport of vesicles or substances from the terminals along microtubules to the nerve cell body (Millecamps and Julien, 2013)] and spread into spinal motoneurons in the CNS through neuromuscular junctions. For instance, PV gains entry into the CNS through receptor-dependent and receptor-independent endocytosis at neuromuscular junctions (Ohka et al., 2004; Ohka et al., 2012). Third, peripheral circulating immune cells can serve as transport vehicles that carry intracellular enteroviruses and pass the CNS through the so-called “Trojan Horse” pathway. Previous studies present that Coxsackieviruses (CVs) migrate to the CNS and traverse the BBB by CV-infected myeloid cells (Tabor-Godwin et al., 2010). Neurotoxic PV can also infect monocytes and exhibits a stronger proliferation ability in these cells (Freistadt and Eberle, 1996). Consequently, a hypothesis has been proposed that monocytes carry PV across CNS (Squires, 1997), although more studies should verify this hypothesis.

**Figure 1:**
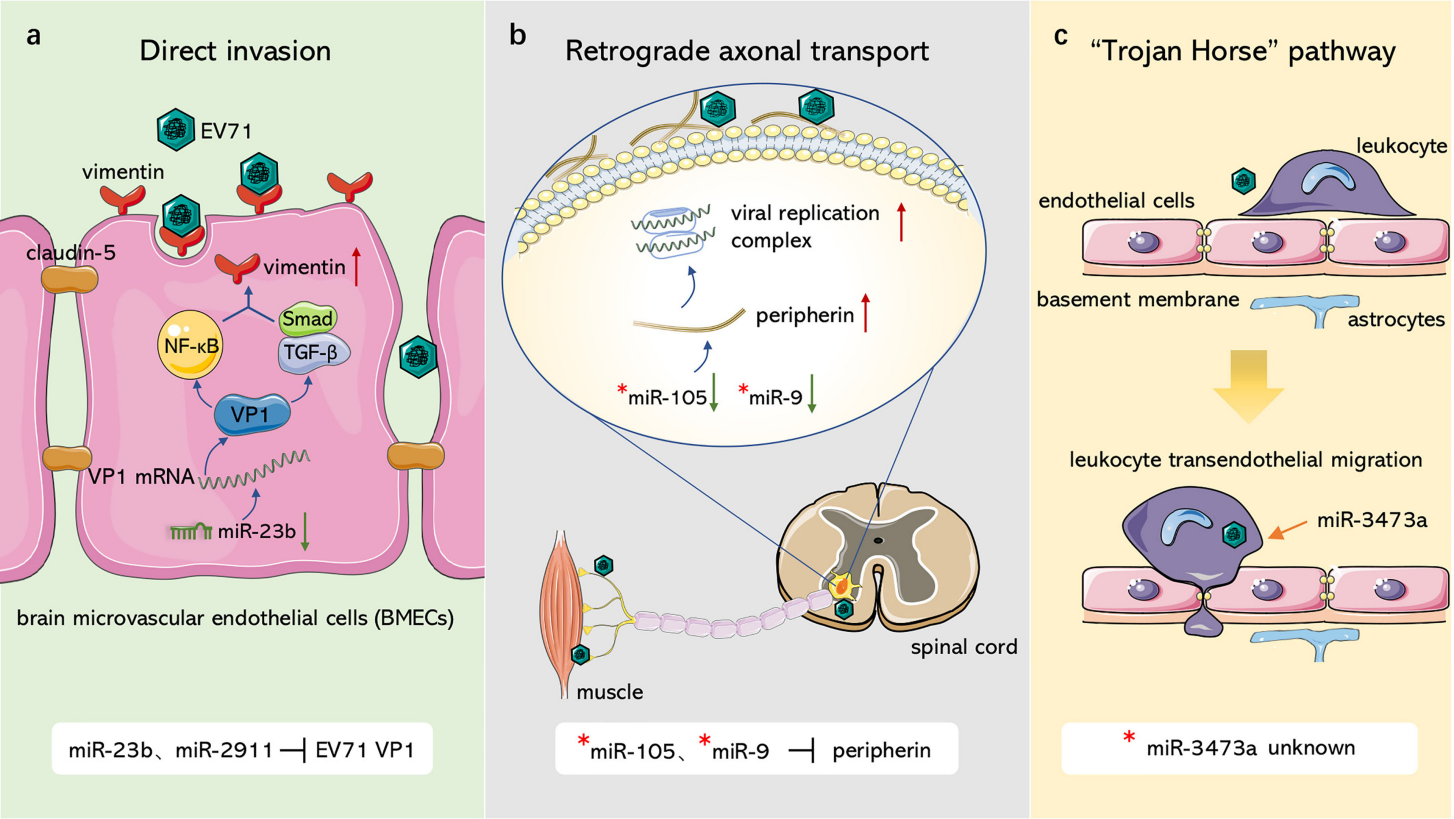
Role of ncRNAs in EV71 invasion through blood-brain barrier. **(A)** Direct invasion. miR-23b and miR-2911 downregulate junction protein claudin-5 and upregulate EV71 receptor vimentin, resulting in damage to blood-brain barrier and the attachment of EV71 through modulating VP1 expression. **(B)** Retrograde axonal transport. miR-105 and miR-9 can target peripherin, which facilitates EV71 attachment and replication, to modulate viral retrograde axonal transport. **(C)** “Trojan Horse” pathway. EV71 can hijack immune cells to intrude CNS, miR-3473a may mediate leukocyte trans-endothelial migration and induce BBB disruption associated with EV71. “*”: non-EV71-infected disease model.

EV71 crosses the BBB and invades the CNS in similar ways as PV, in which miRNAs are involved in regulation of several pathways (**Figure 1**). With regards to the first route, Zhu et al. (Zhu et al., 2019) and Wang et al. (Wang W. et al., 2020) observed that EV71 infected BMECs with the capsid protein, VP1, which reduced claudin-5, the junction protein of endothelial cells, leading to increased BBB permeability and upregulation of the EV71 receptor vimentin to facilitate attachment. miR-2911 and miR-23b mediate neural invasion of EV71 by directly targeting the VP1-coding sequence that regulates VP1 translation (Wen et al., 2013; Li X. et al., 2018). For the second route, Chen et al. (Chen et al., 2007) reported that EV71 infected and entered the CNS through retrograde axonal transport at spinal motor nerves. Lim et al. (Lim et al., 2021) further showed that surface-expressed peripherin in motor neurons provides anchor points for EV71 and contributes to viral transmission, whereas intracellular peripherin modulates EV71 genome replication, resulting in CNS infection. In amyotrophic lateral sclerosis (ALS) patients, miR-105 and miR-9 mainly dominate peripherin expression in motor neurons by targeting the 3’UTR of peripheral mRNA (Hawley et al., 2019); however, their effects on EV71 have not been explored. In EV71-infected mouse neurons, miR-3473a plays a role in axon guidance and Wnt signaling pathways, which control axon growth and guidance (Van Battum et al., 2015) and mediate neuronal positioning as well as axon development (Salinas and Zou, 2008), respectively. Downregulation of miR-3473a activates these two pathways and promotes retrograde axonal transport of EV71 (Yang et al., 2017). For the third route, EV71 was demonstrated to infect human CD14+ cells (Wang J. et al., 2013), leukocytes (Nishimura et al., 2009), dendritic cells (Lin et al., 2009), and other peripheral immune cells, increasing the ability of EV71 to invade the CNS through the Trojan horse pathway. miR-3473a was considered to modulate leukocyte trans-endothelial migration and induce EV71-associated BBB disruption (Yang et al., 2017), however, studies should verify this hypothesis.

## 4 ncRNAs and Nervous System Injury of EV71

Although multiple complications have been reported, brainstem encephalitis with associated neurological pulmonary edema is a characteristic presentation of EV71 CNS infection (Wong et al., 2000; Nolan et al., 2003; Ooi et al., 2010). Affected children develop rapidly progressing cardiopulmonary failure that causes death, which is attributed to respiratory failure and severe pulmonary edema without intensive care. Autopsy and MRI reports indicate that EV71 lesions are mainly located in the ventral, medial, and lateral medulla oblongata (Zimmerman, 1999; Kao et al., 2004). In addition, EV71 has been detected in other nerve tissues, such as the spinal cord, which may explain generation of acute flaccid paralysis.

**Figure 2:**
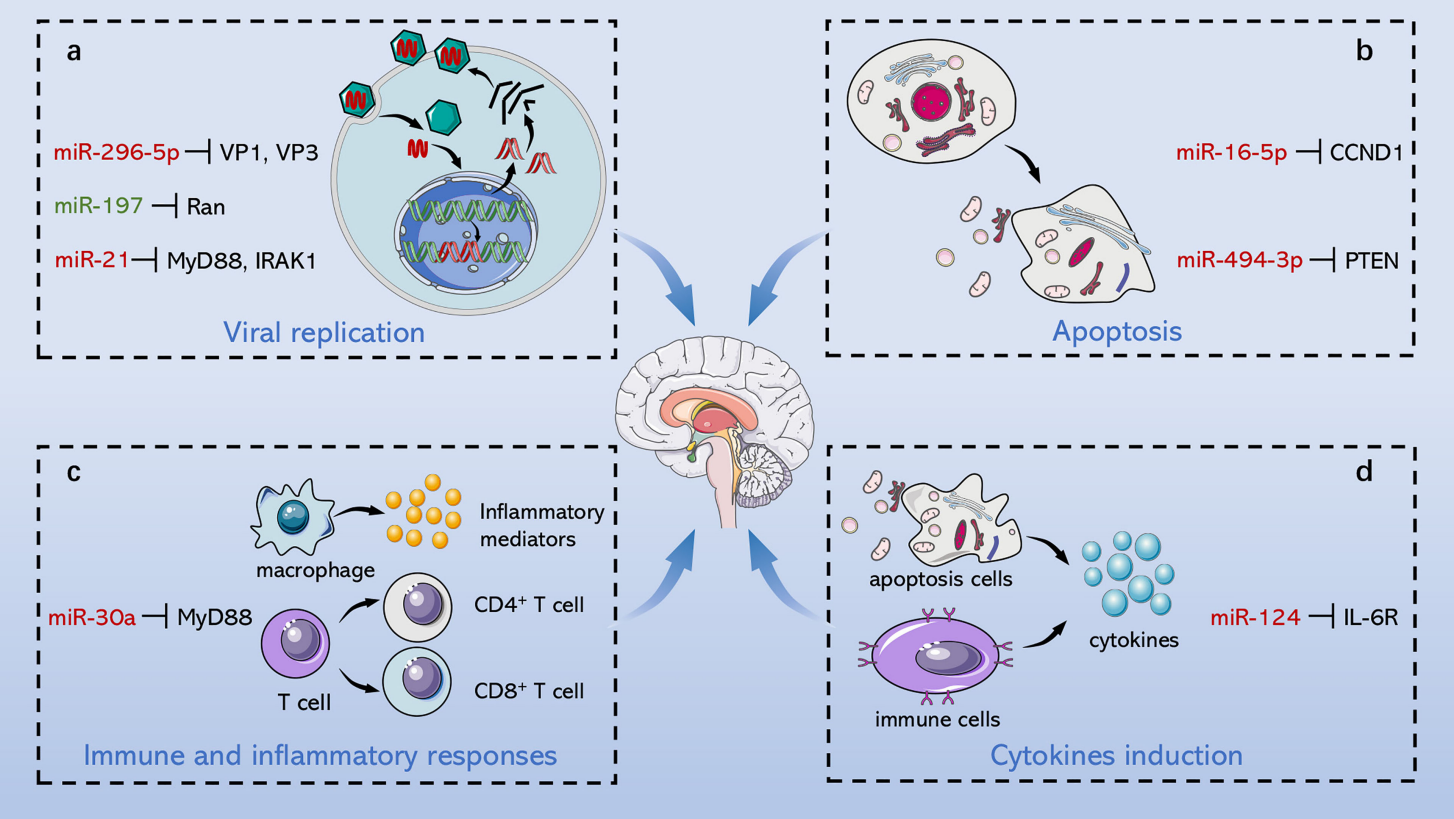
Role of ncRNAs in central nervous system injury of EV71. EV71 injures CNS through direct damage by the virus and indirect injury mediated by immune and inflammatory responses. On the one hand, ncRNAs mediate direct injury of EV71 by modulating viral replication and host apoptosis. **(A)** miR-296-5p, miR-197 and miR-21 separately target viral genome, key host proteins and NF-κB pathway to involve in regulation of viral replication. **(B)** miR-16-5p and miR-494-3p respectively modulate cyclin expression and PI3K/Akt pathway to involve in regulation of host apoptosis. On the other hand, ncRNAs indirectly damage CNS through immunological concomitant inflammatory response and cytokines induction. **(C)** miR-30a promotes CNS damage by regulating immune and inflammatory responses, and **(D)** miR-124 upregulates the key pro-inflammatory cytokine, IL-6, aggravating damage induced by EV71. Red words indicate “upregulation”; green words indicate “downregulation”.

The pathogenesis of EV71-induced neurological complications is caused by host-virus interaction including direct damage by the virus and indirect injury mediated by immune and inflammatory responses (**Figure 2**). Apoptosis is a pivotal process for removing damaged cells and virus-infected cells to resist EV71 infection. Viruses are cytotropic microorganisms that completely rely on the host to survive, and they regulate host cells survival and complete their life cycle by mediating cell apoptosis to facilitate viral translation, replication, assembly, and release. The balance between viral replication and host apoptosis is t key for viral infection and determines direct damage of the virus to the host. On the other hand, the host counters against EV71 infection through innate immune and acquired immune cells, while EV71 escapes immune defense through several pathways, such as intracellular parasitism and immune cell destruction. Inflammatory cells infiltrate brain tissues mediated by virus particles stimulation and immune response, which further releases several inflammatory factors, such as IL-1, IL-6, IL-12, as well as TNF-α; and aggravates nervous system injury.

### 4.1 Direct Damage of EV71 to the Nervous System

#### 4.1.1 Effect of ncRNAs on EV71 Replication

ncRNAs play a crucial regulatory role in various interactions between viruses and their hosts (Esteller, 2011; Beermann et al., 2016). EV71 hijacks host ncRNAs targeting proliferation-related genes of the host or even the virus itself to constitute a microenvironment that promotes EV71 replication. Mechanisms of directly targeting EV71 genome sequence by ncRNAs or modulating key host processes and signaling pathways to inhibit or promote viral replication are presented in this section (**Table 1**).

**Table 1:** ncRNAs involved in EV71 replication.

ncRNAs	Expression	Target	Description	Process	Disease model	Reference
miR-296-5p	up	EV71 VP1 and VP3	miR-296-5p decreases EV71 replication by interacting with viral VP1vand VP3 genes	EV71 replication	*in vitro*: EV71 infected RD and SK-N-SH cells	(Zheng et al., 2013)
miR-373, miR-542-5p	unknown	5’UTR of EV71 genome	miR-373 and miR-542-5p inhibit EV71 replication by targeting 5’-UTR of viral genome	EV71 replication	*in vitro*: EV71 infected RD cells	(Yang and Tien, 2014)
miR-2911	up	EV71 VP1	miR-2911 reduces EV71 replication by directly targeting the VP1-coding sequence	EV71 replication	*in vitro*: EV71(Fuyang-0805 and Lianyungang2015) infected Vero cells	(Li X. et al., 2018)
miR-23b	down	EV71 VP1	downregulated miR-23b advances EV71 replication by targeting the VP1 gene 3’UTR	EV71 replication	*in vitro*: EV71 (Fuyang No. EU703812) infected RD cells	(Wen et al., 2013)
miR-17-5p, miR-19a/b	down	EV71 VP1	downregulated miR-17-5p and miR-19a/b enhance EV71 replication by targeting EV71 gene VP1	EV71 replication	*in vitro*: EV71 (strain FY0805) infected Vero cells	(Fu et al., 2019)
miR-18a, miR-452	up	EV71 VP3	miR-18a and miR-452 decrease EV71 replication by expression inhibition of VP3	EV71 replication	*in vitro*: EV71 (Hubei-09 strain GU434678.1) infected RD cells	(Yang et al., 2021)
29-mer shRNA	:	EV71 3D(pol)	29-mer shRNA most effectively inhibits EV71 replication by targeting EV71 3D(pol)	EV71 replication	*in vitro*: EV71 infected RD cells	(Tan et al., 2007)
miR-127-5p	up	SCARB2	miR-127-5p downregulates the expression of SCARB2 by target SCARB2-coding gene 3’ UTR	EV71 replication	*in vitro*: EV71 (Fuyang0805 strain) infected HeLa and HepG2 cells	(Feng C. et al., 2017)
miR-197	down	Ran	downregulated miR-197 facilitates EV71 replication by suppressing Ran to assist transportation of viral 3D/3C and replication protein	EV71 replication	*in vitro*: EV71 (2231 TW strain) infected HEK 293T and RD cells	(Tang et al., 2016)
miR-134	unknown	Ran	miR-134 represses EV71 replication by decreasing Ran expressions	EV71 replication	*in vitro*: EV71 infected Hep2 and RD cells	(Orr-Burks et al., 2017)
miR-141	up	eIF4E	miR-141 promotes EV71 replication by targeting eIF4E for shutoff of host protein synthesis	EV71 replication	*in vitro*: EV71 infected RD cells	(Ho et al., 2011)
miR-876-5p	up	CREB5	miR-876-5p accelerates EV71 replication by targeting host CREB5	EV71 replication	*in vitro*: EV71 (2231 Taiwan strain) infected RD and SK-N-SH cells	(Xu et al., 2020)
miR-155	up	PICALM	miR-155 inhibits EV71 replication by targeting PICALM	EV71 replication	*in vitro*: EV71 infected RD and SK-N-SH cells	(Wu et al., 2019)
miR-30a	down	Beclin-1	downregulated miR-30a advances EV71 replication by targeting 3’ UTR of Beclin-1 transcripts to inhibit autophagy	EV71 replication	*in vitro*: EV71 infected Hep2 and Vero cells	(Fu et al., 2015)
miR-30a	up	MyD88	miR-30a facilitates EV71 replication by targeting MyD88 and subsequently inhibits IFN-1 production	EV71 replication	*in vitro*: EV71 infected OE cells	(Wang Y. et al., 2020)
miR-548	down	IFN-λ1	downregulated miR-548 decrease EV71 replication by enhancing IFN-λ1 expression	EV71 replication	*in vitro*: EV71 (C4 subtype) infected RD cells	(Li et al., 2013)
miR-155-5p	up	FOXO3, IRF7	miR-155-5p facilitates EV71 replication by negatively regulating FOXO3/IRF7 axis to inhibit IFN-1 response	EV71 replication	*in vitro*: EV71 (BrCr strain) infected RD cells; *in vivo*: EV71 (BrCr strain) infected C57BL/6 mice	(Yang et al., 2020)
lncRNA-AK097647	up	unknown	lncRNA AK097647 facilitates EV71 replication by decreasing IFN-λ1	EV71 replication	*in vitro*: EV71 (BrCr strain) infected RD cells	(Chu et al., 2021)
			lncRNA AK097647 induces the phosphorylation of NF-κB			
miR-526a	down	CYLD	downregulated miR-526a promotes EV71 replication by targeting CYLD to promote the RIG-I-dependent NF-κB pathway	EV71 replication	*in vitro*: EV71 (GDV-103 strain) infected RD cells	(Xu et al., 2014)
miR-9-5p	down	NF-κB	downregulated miR-9-5p promotes EV71 replication by targeting NF-κB and improving its expression	EV71 replication	*in vitro*: EV71 (Shenzhen strain AF30299.1) infected HEK 293T, Vero, RD, HT‐29, HeLa, and THP‐1 cells; *in vivo*: EV71 (Shenzhen strain AF30299.1) infected ICR mice	(Li and Zheng, 2018)
miR-146a	up	TRAF6, IRAK1	miR-146a accelerates EV71 replication by targeting TRAF6 and IRAK1	EV71 replication	*in vivo*: EV71 infected RD cells	(Ho et al., 2014; Fu et al., 2017)
			TRAF6 activates the NF-κB pathway			
miR-545	up	TRAF6	miR-545 advances EV71 replication by attenuating TRAF6 expression	EV71 replication	*in vitro*: EV71 infected HEK 293T and RD cells	(Sun et al., 2019)
miR-628-5p	Up	TRAF3	miR-628-5p promotes EV71 replication by inhibiting TRAF3 expression	EV71 replication	*in vitro*: EV71 infected RD cells	(Li et al., 2021)
miR-21	up	MyD88, IRAK1	miR-21 promotes EV71 replication by targeting MyD88 and IRAK1	EV71 replication	*in vitro*: EV71 infected HCoEpiC and Human NCM460 cells	(Feng N. et al., 2017)
			MyD88 and IRAK1 activate the NF-κB pathway			
miR-124	up	IL-6R, STAT3	miR-124 promotes EV71 replication by restraining the expression of IL-6R and STAT3	EV71 replication	*in vitro*: EV71 infected RD and HeLa cells	(Chang et al., 2017)
miR-302	down	KPNA2	downregulated miR-302 promotes EV71 replication by targeting KPNA2 to regulate the JNK pathway	EV71 replication	*in vitro*: EV71 (Xiangyang strain JN230523.1) infected HEK293T and RD cells	(Peng et al., 2018)
let-7c-5p	up	MAP4K4	MAP4K4 is a key inhibitory factor of the JNK pathway	EV71 replication	*in vitro*: EV71 infected RD cells	(Zhou et al., 2017)
			let-7c-5p remotes EV71 replication by inhibiting MAP4K4 expression			
miR-103, miR-107	down	SOCS3	downregulated miR-103 and miR-107 increase EV71 replication and suppress production of IFN-1 by regulating SOCS3/STAT3 pathway	EV71 replication	*in vitro*: EV71 (BrCr strain) infected Vero and RD cells	(Huang et al., 2021)

The host suppresses EV71 replication through RNA interference of the combination of miRNAs and the viral genome, whereas EV71 downregulates the corresponding miRNAs to circumvent its suppression. miR-296-5p targets EV71 VP1 and VP3 coding sequences (2115 to 2135 nt and 2896 to 2920 nt) and is upregulated in the infected cells. miR-296-5p is a key factor in resisting EV71 infection by preventing synthesis of EV71 VP1/VP3 (Zheng et al., 2013). Several miRNAs, including miR-2911 (Li X. et al., 2018), miR-23b (Wen et al., 2013), and members of the miR-17-92 family (Fu et al., 2019) can also modulate VP1 gene expression of EV71. EV71 downregulates expression of miR-23b, miR-17-5p, and miR-19a/b to strengthen virus invasion and host injury, whereas miR-2911 expression is upregulated by initiation of the antiviral damage system. Yang et al. explored the relationship between miR-373 and miR-542-5p and EV71 replication, and revealed that miR-373 and miR-542-5p directly target the 5’UTR of the viral genome to inhibit EV71 replication (Yang and Tien, 2014). Short hairpin RNAs (shRNAs) have been found to act as therapeutic targets by antagonizing EV71 replication, whereas 29-mer shRNA effectively inhibits EV71 replication by targeting EV71 3D(pol) (Tan et al., 2007). Additionally, ncRNAs target the viral genome to modulate replication of EV71, as well as target receptor-related genes. Further, the EV71 receptor, SCARB2 induces viral infection of cells to directly mediate viral replication and cell tropism (Yamayoshi et al., 2009). miR-127-5p attenuates expression of SCARB2 mRNA and protein (Feng et al.), thus restraining viral internalization and ultimately abrogating virus immune escape. Furthermore, Liu et al. and Sim et al. transfected rhabdomyosarcoma (RD) cells with siRNAs targeting 2Apro (Liu et al., 2016), 3’UTR, 2C, 3C, and 3D (Sim et al., 2005) region of EV71 genome separately, significantly decreasing cytopathic effects of EV71 through RNA interference. These findings indicate that ncRNAs are potential therapeutic targets for preventing viral infection and alleviating body injury.

ncRNAs are involved in key processes and signaling pathways to modulate viral biosynthesis. The nuclear protein Ran affects several significant cellular processes, including the regulation and control of cell cycle progression by mediating mitosis, and nucleocytoplasmic transport associated with Ran GTPase (Dasso, 2001; Clarke and Zhang, 2008). EV71-induced miR-197 (Tang et al., 2016) and miR-134 (Orr-Burks et al., 2017) target Ran gene, which assists nuclear transportation of viral proteins 3D/3C and replication-associated proteins, ultimately dampening EV71 replication. The life cycle of viruses is dependent on the host translation machinery, whereby cap-dependent protein translation is beneficial to the host whereas cap-independent translation is beneficial to the virus. Notably, it is evident that degradation of eukaryotic initiation factor 4E (eIF4E) determines the progress of the switch between the two translation processes (Richter and Sonenberg, 2005; Sukarieh et al., 2010). Elsewhere, Ho et al. found that the eIF4E gene is combined and cleaved by upregulated miR-141 resulting in shutoff of the host protein synthesis and generation of viral proteins (Ho et al., 2011).

**Figure 3:**
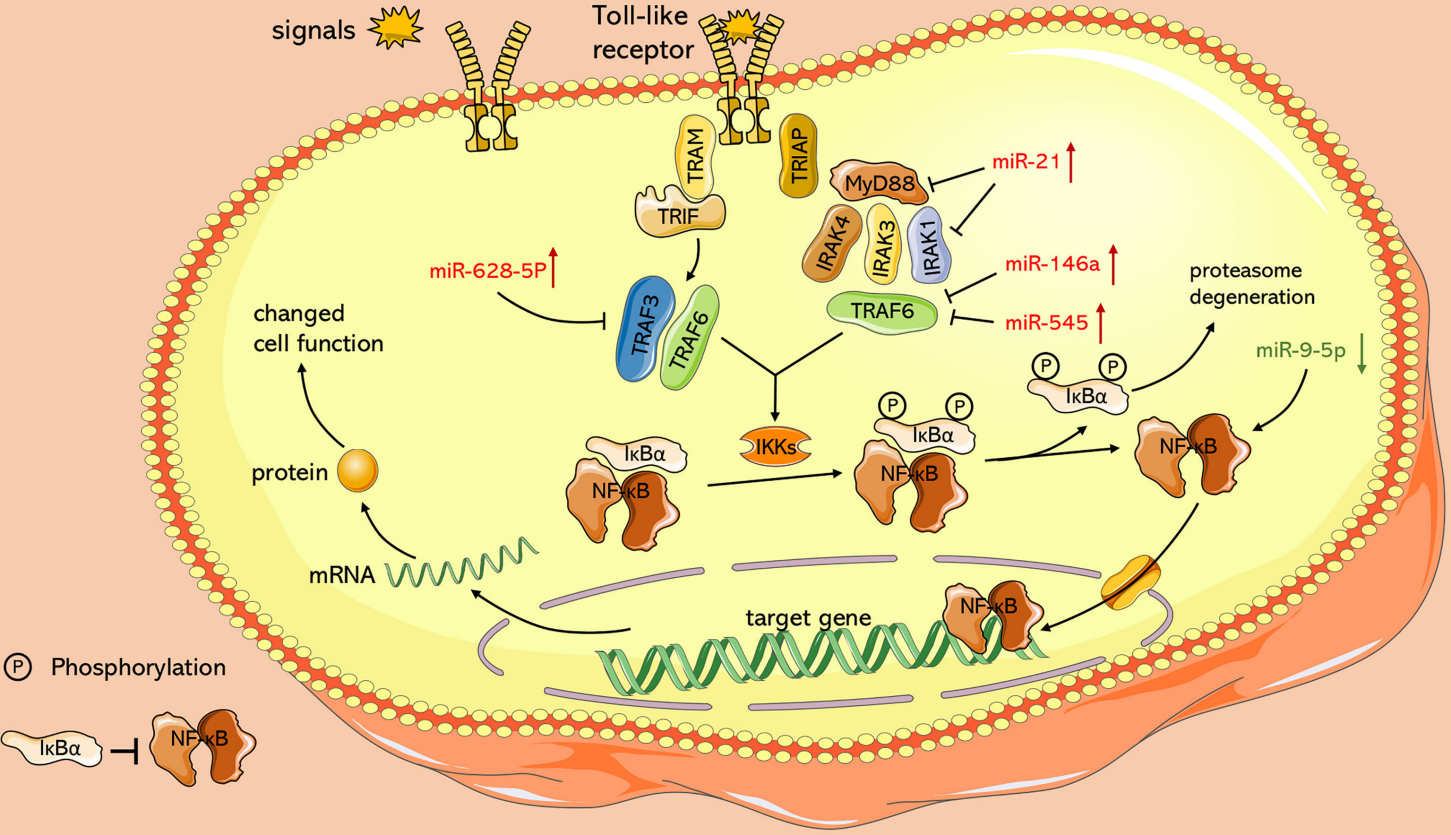
Role of ncRNAs in activation of NF-κB pathway with TLR signaling as an example. Toll-like receptors activate and recruit TIR-containing adaptor molecules, MyD88 and TRIF, which prime downstream effectors respectively, under the stimulation of signals such as LPS. Both of upstream signal paths finally transfer signals to IKKs, the protein kinase of IκB, and relieve inhibition of NF-κB. miR-628-5p, miR-21, miR-146a, miR-545 and miR-9-5p play an essential role in this process.

In addition, it has been found that ncRNAs are associated with dysregulated signaling pathways (**Figure 3** **)**. The nuclear factor-kappa B (NF-κB) pathway regulates several genes related to cell proliferation, differentiation, innate immune response, and inflammatory cytokine production (Perkins, 2012). NF-κB pathway plays an essential role in EV71 pathogenicity, which is represented by the viral 2C protein, which suppresses NF-κB pathway activation to promote viral replication (Tung et al., 2010; Du H. et al., 2015). Myeloid differentiation factor 88 (MyD88) and IL-1 receptor-associated kinase-1 (IRAK1) modulate initiation of the Toll-like receptor-dependent NF-κB pathway (Hayden et al., 2006). According to Feng N. et al. (2017), it is demonstrated that miR-21 promotes EV71 replication by suppressing the NF-κB pathway mediated by MyD88 and IRAK1. Moreover, other studies including Ho et al. (2014) and Fu et al. (2017) confirmed that miR-146a downregulates expression of TNF receptor-associated factor 6 (TRAF6), which regulates activation of NF-κB pathway (Xia et al., 2011), and hence facilitates viral biosynthesis. However, downregulated miR-526a (Xu et al., 2014) and miR-9-5p (Li and Zheng, 2018) induced by EV71 infection facilitate EV71 replication by activating the NF-κB pathway. These conflicting results may be ascribed to multiple functions of NF-κB in different cases. Under physiological conditions, NF-κB mediates immune response to resist external invasion, whereas aberrant regulation of NF-κB is implicated in cancer development and EV71 pathogenicity (Baud and Karin, 2009; Tung et al., 2010; Jin et al., 2018). In addition, Chu et al. reported that lncRNA-AK097647 has been found significantly upregulated during EV71 infection, which facilitates EV71 replication through blocking interferon-λ1 secretion and inducing the phosphorylation of NF-κB (Chu et al., 2021).

In conclusion, these findings indicate that ncRNAs regulate viral replication by targeting key virus and host genes. In the future, we can target these ncRNAs to inhibit EV71 replication and reduce body injury.

#### 4.1.2 ncRNAs and Host Apoptosis Induced by EV71

Host injury affected by viruses is influenced by apoptosis. Viruses regulate the host apoptosis to complete their replication cycle, whereas virus-infected host cells initiate apoptotic pathways to resist viral infection and reduce virus damage on the body (Benedict et al., 2002; Orzalli and Kagan, 2017). The endogenous mitochondrial cytochrome C pathway and exogenous death receptor Fas/FasL pathway are the key pathways in controlling cell apoptosis. The cascade of caspase protease family activation, which is the mutually terminal process of the two pathways, contributes to decomposition of potentially harmful cells (Sen, 1992; Riedl and Shi, 2004; Green and Llambi, 2015). ncRNAs control EV71-related cell apoptosis by regulating protein expression and signal transduction of the caspase pathway (**Table 2**).

**Table 2:** ncRNAs involved in host apoptosis.

ncRNAs	Expression	Target	Description	Process	Disease model	Reference
miR-16-5p	up	CCND1, CCNE1	miR-16-5p promotes host apoptosis by targeting CCNE1 and CCDN1	Apoptosis	*in vitro*: EV71 (GZ-CII strain) infected RD, CCF-STTG1 and SK-N-SH cells; *in vivo*: KM and ICR mice	(Zheng et al., 2017)
miR-let-7b	up	CCND1	miR-let-7b promotes host apoptosis by inhibiting CCND1 expression	Apoptosis	*in vitro*: EV71 infected SH-SY5Y cells	(Du X. et al., 2015)
miR-146a	up	SOS1	miR-146a promotes EV71-induced host apoptosis by targeting 3’UTR of SOS1 gene	Apoptosis	*in vitro*: EV71 infected RD cells	(Chang et al., 2015)
			SOS1 accelerates cell apoptosis			
miR-370	down	GADD45β	downregulated miR-370 advances EV71-induced apoptosis by targeting GADD45β	Apoptosis	*in vitro*: EV71 infected RD cells	(Chang et al., 2015)
lnc-IRAK3-3	up	miR-891b	lnc-IRAK3-3 restrain the expression of miR-891b to promote host apoptosis	Apoptosis	*in vitro*: EV71 infected RD cells	(Liao et al., 2019)
miR-891b	down	GADD45β	miR-891b is inhibited by lnc-IRAK3-3 downregulated miR-891b increases host apoptosis by raising GADD45β generation	Apoptosis	*in vitro*: EV71 infected RD cells	(Liao et al., 2019)
miR-874	down	GZMB	downregulated miR-874 facilitates host apoptosis by reducing GZMB expression	Apoptosis	*in vitro*: EV71 infected Jurkat cells	(Zhang M. et al., 2020)
miR-27a	down	EGFR	downregulated miR-27a inhibits host apoptosis by enhancing EGFR expression and initiating PI3K/AKT pathway	Apoptosis	*in vitro*: EV71 infected RD and SK-N-SH cells	(Zhang et al., 2014)
			The activation of the PI3K/AKT pathway suppresses host apoptosis			
miR-494-3p	up	PTEN	miR-494-3p inhibits host apoptosis by targeting PTEN and initiating PI3K/Akt signaling pathway	Apoptosis	*in vitro*: EV71 infected RD and HEK 293T cells	(Zhao et al., 2018)
			PTEN is an inhibitor of the PI3K/AKT pathway			
miR-545	up	TRAF6, PTEN	miR-545 inhibits host apoptosis by attenuating PTEN expression	Apoptosis	*in vitro*: EV71 infected RD and HEK 293T cells	(Sun et al., 2019)
lncRNA- MALAT1	up	miR-194-5p	lncRNA- MALAT1 induces host apoptosis by MALAT1/miR-194-5p/DUSP1 ceRNA regulatory axis	Apoptosis	*in vitro*: EV71 (87-2008 Xi’an Shaanxi strain) infected RD cells	(Lu et al., 2021)

Cyclin D1 (CCND1) and cyclin E1 (CCNE1) are the main regulators of G1 phase progression (Blomen and Boonstra, 2007). Notably, miR-16-5p (Zheng et al., 2017), and miR-let-7b (Du X. et al., 2015) abrogate EV71 replication through inhibition of CCND1 synthesis and initiation of caspase-dependent apoptosis. Furthermore, endogenous miR-let-7b released from injured neurons can induce neuronal cell death through Toll-like receptor (TLR) 7 signaling (Lehmann et al., 2012). Son of sevenless homolog 1 (SOS1) is a critical anti-apoptotic protein associated with TNFα-induced apoptosis (Kurada et al., 2009; Hao et al., 2018), Growth arrest and DNA damage-inducible protein 45β (GADD45β) promotes apoptosis by upregulating expression of the apoptosis-related factors, caspase-3 and p53 (Ou et al., 2010; Yu et al., 2013). Elsewhere, Chang et al. (Chang et al., 2015) reported that induction of miR-146a and degradation of miR-370 together trigger apoptosis of the EV71-infected cells by targeting SOS1 and GADD45β, respectively. Moreover, the long non-coding RNA, lnc-IRAK3-3 was found to capture miR-891b upregulate GADD45β expression and eventually promote host apoptosis (Liao et al., 2019). Moreover, Lu et al. filtered differentially expressed ncRNAs associated with EV71 infection, including 6 lncRNAs, 28 miRNAs, and 349 mRNAs. Their further studies reported that MALAT1/miR-194-5p/DUSP1 axis, a lncRNA-miRNA-mRNA-associated competing endogenous RNA regulatory network, involved in host apoptosis induced by EV71 infection (Lu et al., 2021).

Phosphatidylinositide 3-kinase (PI3K)/Akt is an important signaling pathway that mediates cell survival, growth, and metabolism (Osaki et al., 2004; Soulard and Hall, 2007; Ediriweera et al., 2019), This pathway attenuates cell apoptosis by inhibiting phosphorylation of caspase-9 and Bad protein (members of the B-cell lymphoma-2 family) (She et al., 2005; Hohenester et al., 2010). Phosphatase and tensin homologue (PTEN) facilitates dephosphorylation of Akt and hence prevents the events of downstream signaling that are regulated by Akt, and thus it is a negative regulator of the PI3K/Akt pathway (Song et al., 2012). The function of PTEN in inhibiting cell apoptosis has been shown in multiple cell types, including kidney cancer cells, mouse mammary epithelia and B lymphocytes (Dupont et al., 2002; Lin et al., 2007; Cheng et al., 2009). Notably, miR-494-3p expression is significantly upregulated following EV71 infection, repressing host apoptosis and promoting EV71 replication through degeneration of PTEN. Overexpression of miR-494-3p mimics antagonizes this process by restoring miR-494-3p levels of expression and activating the PI3K/Akt pathway (Zhao et al., 2018). miR-545 separately targets PTEN and TRAF6 and activates PI3K/Akt and NF-κB pathways to modulate EV71 replication and host apoptosis (Sun et al., 2019). Epidermal growth factor receptor (EGFR) is an activator of the PI3K/Akt pathway (Guo et al., 2015), and is upregulated by EV71-induced downregulation of miR-27a and eventually inhibits nerve cell apoptosis (Zhang et al., 2014).

These findings indicate that hosts and viruses fight for damage and anti-damage around viral replication and host apoptosis, which are regulated by ncRNAs. These ncRNAs can serve as therapeutic targets to inhibit viral life cycle and alleviate host injury in EV71 treatment.

### 4.2 Indirect Injury Mediated by Immune and Inflammatory Responses

Although inflammation is a protective response to minimize pathogen spread and promote the recovery of damaged tissue, a dysregulated inflammatory response results in various inflammatory injuries (Nathan and Ding, 2010). Immunological concomitant inflammatory injury is the major damage mode of EV71 to hosts with severe nervous system diseases such as neurogenic pulmonary edema (Lin et al., 2003; Huang et al., 2011; Griffiths et al., 2012; Yu et al., 2019). A complex immune defense mechanism is triggered when EV71 infects the body, which is accompanied by resistance mediated by the innate immune response to exogenous pathogens and activation of acquired immune response mediated by antigen-presenting cells (Edsall, 1949). Subsequently, the activated immune-related cells (innate immune cells and specific immune cells) release cytokines and inflammatory factors, which may cause immune-mediated inflammatory injury in pathological conditions. Effects of ncRNAs on immune and inflammatory responses as well as cytokine expression are discussed in the subsequent section (**Table 3**).

**Table 3:** ncRNAs involved in immune and inflammatory responses.

ncRNAs	Expression	Target	Description	Process	Disease model	Reference
miR-21	up	MyD88, IRAK1	miR-21 reduces the production of IFN-1 by targeting MyD88 and IRAK1	pro-inflammatory factor	*in vitro*: EV71 infected HCoEpiC and Human NCM460 cells	(Feng N. et al., 2017)
miR-30a	up	MyD88	miR-30a reduces the production of IFN-1 by targeting MyD88 and IRAK2	pro-inflammatory factor	*in vitro*: EV71 infected OE cells	(Wang Y. et al., 2020)
miR-526a	down	CYLD	miR-526a rises the level of IFN-1 through the RIG-I-dependent pathway	anti-inflammatory factor	*in vitro*: EV71 (GDV-103 strain) infected RD cells	(Xu et al., 2014)
miR-9-5p	down	NFκB	miR-9-5p inhibits excessive production of IL-6, IL-1β, and TNF-α induced by EV71	anti-inflammatory factor	*in vitro*: EV71 (Shenzhen strain AF30299.1) infected HEK 293T, Vero, RD, HT‐29, HeLa, and THP‐1 cells; *in vivo*: EV71 (Shenzhen strain AF30299.1) infected ICR mice	(Li and Zheng, 2018)
			miR-9-5p increases production of IFN-1 by targeting NFκB			
miR-146a	up	IRAK1, TRAF6	miR-146a reduces the expression of IFN-β by targeting IRAK1 and TRAF6	pro-inflammatory factor	*in vivo*: EV71 infected RD cells	(Ho et al., 2014; Fu et al., 2017)
miR-155-5p	up	FOXO3, IRF7	miR-155-5p inhibits IFN-1 response by negatively regulating the FOXO3/IRF7 axis	pro-inflammatory factor	*in vitro*: EV71 (BrCr strain) infected RD cells; *in vivo*: EV71 (BrCr strain) infected C57BL/6 mice	(Yang et al., 2020)
miR-545	up	PTEN, TRAF6	miR-545 inhibits IFN-1 generation by attenuating TRAF6 and PTEN expression	pro-inflammatory factor	*in vitro*: EV71 infected HEK 293T and RD cells	(Sun et al., 2019)
miR-628-5p	up	TRAF3	miR-628-5p inhibits IFN-β expression by targeting TRAF3	pro-inflammatory factor	*in vitro*: EV71 infected RD cells	(Li et al., 2021)
miR-103, miR-107	down	SOCS3, STAT3	miR-103 and miR-107 advance the level of IFN-1 by targeting SOCS3	anti-inflammatory factor	*in vitro*: EV71 (BrCr strain) infected Vero and RD cells	(Huang et al., 2021)
miR-124	up	IL-6R, STAT3	miR-124 enhances the level of IL-6 by targeting IL-6R	pro-inflammatory factor	*in vitro*: EV71 infected RD and HeLa cells	(Chang et al., 2017)
miR-302	down	KPNA2	KPNA2 overexpression promotes EV71-induced production of the IL-6 and TNF-α	anti-inflammatory factor	*in vitro*: EV71 (Xiangyang strain JN230523.1) infected HEK 293T and RD cells	(Peng et al., 2018)
			miR-302 inhibits the expression of KPNA2 mRNA and protein			
let-7c-5p	up	MAP4K4	MAP4K4 is a key inhibitory factor of the JNK pathway let-7c-5p promotes IL-6 and TNF-α by inhibiting MAP4K4 expression	pro-inflammatory factor	*in vitro*: EV71 infected RD cells	(Zhou et al., 2017)

#### 4.2.1 Role of ncRNAs in Immune and Inflammatory Responses During EV71 Infection

Innate immune system serves as the first line of defense against exogenous pathogens and internal apoptosis, aberrant cells, and other “nonself” components. Chemokines and cytokines are released and inflammatory response is initiated after rapid activation of innate immune cells through recognition of foreign or harmful substances. Recognition of viruses is primarily initiated by pattern recognition receptors (PRRs), including Toll-like receptors (TLRs), retinoic acid inducible-gene I (RIG-I)-like receptors, NOD-like receptors (NLRs), and C-type lectin receptors (Takeuchi and Akira, 2010; Fitzgerald and Kagan, 2020). Serving as a key target of viruses against body immunity, MyD88 is an essential adaptor molecule for TLR signaling cascades (Takeda and Akira, 2004). Research conducted by Wang et al., illustrated that overexpression of miR-30a upon EV71 infection inhibited innate immunity by repressing type I interferon production, and the direct target of miR-30a, MyD88, played a key role in this process (Wang Y. et al., 2020). Analogously, miR-21 targets MyD88 and IRAK1 to reduce the level of type I interferon through the TLR pathway (Feng N. et al., 2017). In addition, Xu et al. reported that RIG-I activity is mediated by miR-526a through inhibition of CYLD expression, which negatively regulates generation of type I interferon (Xu et al., 2014). Overexpression of miR-9-5p which is induced by EV71 also inhibits RIG-I-dependent innate immune response by targeting NF-κB (Li and Zheng, 2018). These ncRNAs control occurrence of inflammatory response by mediating innate immunity, while the recognition receptors of innate immune cells, PRRs, are involved in transcriptional regulation of inflammatory mediators (Takeuchi and Akira, 2010; Luo et al., 2019). For instance, overexpression of pro-inflammatory cytokines (TNF-α, IL-6, and IL-1) induced by EV71 is restored through modulation of RIG-associated miR-9-5p (Li and Zheng, 2018). In innate immune response, macrophages play an important role in the initiation, maintenance and dissipation of inflammation, and Early Growth Response 1 (EGR1) inhibits expression of pro-inflammatory genes in macrophages (Trizzino et al., 2021). Hu et al. evaluated the relationship of circRNA/miRNA/mRNA associated with EV71 infection, and eventually screened hsa_circ_0017115/hsa-miR-150-5p/EGR1 axis (Hu et al., 2021), which might regulate inflammatory response through interaction between EGR1 and macrophages.

Acquired immune cells are activated by stimulation of antigen signals and play an essential role in resisting infections (Edsall, 1949). Chang et al. (2006) and Yang et al. (2001) found that cellular rather than humoral immunity is associated with host acquired immune response against EV71 infection. Dicer serves as an RNase III enzyme and modulates production of mature miRNAs (Burger and Gullerova, 2015). Knockout of Dicer during the early stage of lymphocyte development shows that miRNAs play a key role in T-cell proliferation as indicated by a 90% reduction in T cells in circulation (Cobb et al., 2005). Moreover, miRNAs are implicated in production of CD4+ Treg cells and Th2 cells late T cell differentiation (Muljo et al., 2005). Previous studies conducted by Thai et al. (2007) and Rodriguez et al. (2007) discovered that miR-155 knockout mice were unable to mount an effective acquired immune response and showed a selective tendency towards Th2 phenotype. Subsequent studies established that miR-155 was conducive to Treg development by targeting suppressor of cytokine signaling 1 (SOCS1) (Lu et al., 2009). Furthermore, Liu et al. (2008) showed that miR-181a induces CD4+ and CD8+ double-positive (DP) T cell development. Th1 cells mainly produce IFN-γ, which has been proven to be a pro-inflammatory factor (Farrar and Schreiber, 1993). Moreover, Th2 and Treg cells mainly produce IL-10, which serves as an anti-inflammatory factor and prevents excessive tissue disruptions caused by inflammation (Ouyang et al., 2011). These results indicate that ncRNAs contribute to inflammation by releasing inflammatory factors through regulation of T cell differentiation and initiating T lymphocytes. However, studies have not explored the effects of ncRNAs on T-cell development and differentiation in EV71-induced disease models.

#### 4.2.2 Role of ncRNAs in Expression of EV71-Induced Cytokines

Abundant cytokines and chemokines are released from activated immune and apoptotic cells induced by EV71, including IFNs, IL-6, IL-13, IL-1β, and TNF-α among other inflammatory mediators (Huang et al., 2011; Ouyang et al., 2011; Griffiths et al., 2012; Luo et al., 2019; Yu et al., 2019). Pro-inflammatory cytokines (such as IL-6, IL-12, IL-1β, TNF-α, and IFN-γ) play a significant role in EV71-mediated CNS inflammatory injury (Lin et al., 2003). The key pro-inflammatory mediator, IL-6, is the main pathogenic factor for pulmonary edema-associated encephalitis (Lin et al., 2002; Luo et al., 2019). Further, the ncRNAs modulate expression of inflammatory cytokines during EV71 infection. A separate study conducted by Chang et al. (Chang et al., 2017) found that miR-124 decreased the level of IL-6 by directly targeting IL-6R and hence promoted EV71 pathogenesis. Moreover, let-7c-5p, which also acts as a pro-inflammatory factor, increases production of IL-6 and TNF-α through the MAP4K4-mediated c-Jun N-terminal kinase (JNK) pathway (Zhou et al., 2017). Analogously, miR-302 has exhibited an anti-inflammatory function by inhibiting EV71-induced generation of IL-6 and TNF-α to alleviate body damage through the miR-302/karyopherin α2 (KPNA2) axis associated with the JNK pathway (Peng et al., 2018). An investigation conducted by Li et al. reported that excessive production of IL-6, IL-1β, and TNF-α was transferred to physiological levels by anti-inflammatory factor miR-9-5p through modulation of the RIG-I-dependent NF-κB pathway (Li and Zheng, 2018). In addition, several ncRNAs, such as miR-103, miR-107 (Huang et al., 2021), miR-146a (Ho et al., 2014; Fu et al., 2017), miR-155-5p (Yang et al., 2020), miR-545 (Sun et al., 2019), and miR-628-5p (Li et al., 2021) negatively or positively affect inflammatory response by modulating expression of interferons. Accordingly, EV71 facilitates inflammatory injury by upregulating pro-inflammatory factors such as miR-124 and downregulating inflammatory factors such as miR-302. Many researchers have reported the role of lncRNAs in secretion of enteroviruses-mediated inflammatory factors. In Coxsackievirus B3 infection, Cao et al. found that lncRNA HIF1A-AS1 activated NF-κB pathway by targeting miR-138, and presented a role of pro-apoptosis and pro-inflammation (Cao et al., 2020). However, EV71-associated lnRNAs in immune and inflammatory responses have not been clarified, more attention should be paid to lncRNAs because of their important potential.

## 5 Potential Clinical Application of ncRNAs in HFMD

### 5.1 ncRNAs and HFMD Diagnosis

Additional laboratory tests are generally deemed unnecessary for mild cases of HFMD because it is a self-limiting disease (Cox and Levent, 2018). However, classification of pathogenic enterovirus and definitive therapy is crucial in the presence of severe or fatal neurological complications associated with HFMD. For instance, the early clinical symptoms of EV71 and CVA16 (the two main pathogens of HFMD) are similar, but few patients with EV71 infection may progress into serious CNS complications; nevertheless, most patients with CVA16 infection show good prognosis (Liu et al., 2015). The golden criterion for diagnosis of enterovirus infection is isolation of viruses from clinical samples, which is time-consuming and laborious (Ooi et al., 2010). Quantitative real-time PCR (qRT–PCR) is a fast method which is developed to circumvent the limitations of conventional diagnostic methods. However, the method is associated with a high number of false-positive and false-negative results owing to the high rate of gene mutation in enterovirus (Perera et al., 2004; Chen et al., 2006). Furthermore, the ncRNAs have highly stable physical and chemical properties during circulation; and thus they can rapidly and conveniently provide an alternative diagnosis strategy for HFMD (Grasedieck et al., 2012; Zhang et al., 2019).

Accumulating studies indicate that ncRNAs can serves as potential candidate biomarkers for the diagnosis of HFMD. A miRNA-based prediction model of HFMD was established by Min et al. (Min et al., 2018), results of the study showed that circulating salivary hsa-miR-221 was continuously and significantly downregulated in all HFMD cohorts. The detection of circulating salivary hsa-miR-221 could be used as a convenient diagnostic method for HFMD. Li Y. et al. (2018) analyzed lncRNA and mRNA expression profiles associated with EV71 infection, and 23 lncRNAs and 372 mRNAs with remarkable differential expression were found between infected and uninfected RD cells. Subsequent studies discovered that these lncRNA were involved in EV71 infection-induced pathogenesis. Additionally, ncRNA can be used to differentiate HFMD caused by EV71 and CVA16, providing a basis for clinical treatment. Cui et al. (2011) reported that miR-545, miR-324-3p, and miR-143 can be used to effectively distinguish EV71 and CVA16 infections in patients with HFMD. Moreover, an investigation conducted by Liu et al. (2020) indicated that patients with EV71-induced HFMD presented significantly higher levels of serum miR-494 as compared with the level in healthy people or those with CVA16-induced HFMD, showing its potential diagnostic value. In addition, ncRNAs play an important role in prediction of disease severity. Meng et al. analyzed the dynamic differential expression profile of lncRNAs and filtered out 10 lncRNAs that were differentially expressed in patients with HFMD presenting with different severities (Meng et al., 2017). Similarly, the comparison of miRNA expression profiles between patients with mild and severe HFMD shows that miR-671-5p, miR-16-5p, and miR-150-3p are potential diagnostic markers for differentiating severity of HFMD (Jia et al., 2014). Furthermore, the level of miR-876-5p is 9.5-fold higher in severe cases than level in cases with mild EV71 symptoms, and the clinical symptoms were alleviated after knockdown of miR-876-5p (Wang et al., 2016). In addition, there are ncRNAs serving as biomarkers for HFMD caused by other non-EV71 and non-CVA16 enteroviruses. Coxsackievirus B5 (CVB5) is a major pathogen of HFMD, which has an increasing incidence in recent years. Teng et al. analyzed the lncRNA profile of CVB5 infected RD and SH−SY5Y cells through RNA sequencing, and revealed the potential of lncRNA-IL12A as a biomarker (Teng et al., 2022). These studies indicate that ncRNAs have significant potential for application in clinical diagnosis of HFMD.

### 5.2 ncRNAs and Treatment of EV71-Induced HFMD

Several studies have explored the potential of ncRNAs as therapeutic targets for treatment of the disease. Studies on miRNAs have achieved positive results and miRNAs are applied in clinical practice (Lee et al., 2020). Novel antiviral drugs have been developed by mainly modulating the function of miRNAs to enhance their roles through mimics and downregulate their roles through inhibition using antisense oligonucleotides (ASOs) (Beermann et al., 2016). As mentioned above, ncRNAs modulate progression of EV71-induced HFMD by regulating the viral life cycle and host immune and inflammatory responses; therefore, these processes can serve as potential therapeutic targets. The miRNAs such as miR-296-5p, miR-197, miR-16-5p, and miR-27a inhibit EV71 proliferation and reduce host injury by modulating viral replication and host apoptosis, respectively. The miRNA analogues can be designed for treatment of EV71-induced HFMD. In addition, inhibitors of proinflammatory factors such as miR-21, miR-146a, and miR-124 can modulate immune response and hence relieve inflammatory injury caused by EV71. Clinical trials of miRNAs based on therapy in cancers are underway implying that miRNA-based therapy for HFMD may be realized in the future (Janssen et al., 2013; van Zandwijk et al., 2017).

However, some limitations were noted in the current study, several of which should be solved before miRNAs can be applied in clinical practice. First, some miRNAs, such as the miR-143/145 cluster (Dimitrova et al.) mentioned above, exhibit opposite effects under different conditions, and thus unified standards of the disease model should be determined. Second, it has been noted that one miRNA can target several genes, whereas one gene can be targeted by several miRNAs. Studies should explore strategies to ensure that miRNAs act on desired targets and to minimize side effects. Third, miRNAs function in multiple organs of the whole body, and the blood-brain barrier blocks entry of most pathogens and drugs. Methods for facilitating miRNA-targeted transport to the brain and across the blood-brain barrier should be explored. Finally, stability of mRNAs should be improved and rapid degeneration of miRNAs should also be minimized. Therefore, further studies should be conducted to explore the role and mechanism of miRNAs in HFMD induced by EV71.

## 6 Conclusions and Perspective

As the main pathogen of HFMD with severe neurological complications, EV71 significantly does harm to patient health and results in a huge economic burden. Therefore, explore neuropathogenic mechanism of EV71 is necessary for reducing severe cases and for development of effective therapeutic ways. We illustrate recent advances concerning the role of ncRNAs in EV71-induced CNS infection and CNS injury by virus-host interaction. As the essential molecules of gene regulation, ncRNAs present broad clinical application prospects. Especially in diagnosis of HFMD, the different expression of ncRNAs have potential in prediction of disease severity and differentiation of HFMD. In conclusion, ncRNAs are closely related to EV71-induced infection progression and virus-host interaction, as well as represent a significant potential direction for therapeutic and diagnostic research. Among them, miRNAs were widely reported in regulation of EV71 life cycle and host immune response. However, although lncRNAs have been shown to participate in viral replication, host apoptosis, and immune and inflammatory responses in enteroviruses infection (Shi et al., 2016; Liu et al., 2019; Zhang Y. et al., 2020), current research on the role of lncRNAs in EV71 infection is limited. It has been shown that lncRNAs may be equally or even more important compared with miRNAs in terms of clinical benefits owing to their tissue specificity. Therefore, there is still need for further studies to explore role of lncRNAs in pathogenesis, diagnosis and treatment of EV71 is necessary.

## Author Contributions

Conceptualization, FY, NZ, YD, and JM; resources, YC; writing—original draft preparation, JY and XC; writing—review and editing, MX and RM. All authors have read and agreed to the published version of the manuscript.

## Funding

This work was funded by the National Natural Science Foundation of China (No. 31701162) and the Key Research and Development Program of Anhui Province (No. 202104a07020031).

## Conflict of Interest

The authors declare that the research was conducted in the absence of any commercial or financial relationships that could be construed as a potential conflict of interest.

## Publisher’s Note

All claims expressed in this article are solely those of the authors and do not necessarily represent those of their affiliated organizations, or those of the publisher, the editors and the reviewers. Any product that may be evaluated in this article, or claim that may be made by its manufacturer, is not guaranteed or endorsed by the publisher.
